# Determinants of drug-resistant tuberculosis in Hunan province, China: a case-control study

**DOI:** 10.1186/s12879-024-09106-5

**Published:** 2024-02-13

**Authors:** Temesgen Yihunie Akalu, Archie C. A. Clements, Zuhui Xu, Liqiong Bai, Kefyalew Addis Alene

**Affiliations:** 1 School of Population Health, Faculty of Health Sciences, Curtin University, Perth, Western Australia Australia; 2 Geospatial and Tuberculosis Research Team, Telethon Kids Institute, Perth, Western Australia Australia; 3 Institute of Public Health, College of Medicine and Health Sciences, University of Gondar, Gondar, Ethiopia; 4 Penninsula Medical School, University of Plymouth, Plymouth, UK; 5 Xiangya School of Public Health, Central South University, Changsha, China; 6 TB Control Institute of Hunan Province, Changsha, China

**Keywords:** Case-control, Determinants, Hunan Province, Drug-resistant tuberculosis

## Abstract

**Background:**

Drug-resistant tuberculosis (DR-TB) is a major public health threat in Hunan Province, with an increasing clinical burden in recent years. This study aimed to identify socio-demographic and clinical factors associated with DR-TB in Hunan province, China.

**Methods:**

A case-control study was conducted in Hunan province. Cases were all DR-TB patients who were confirmed by culture and Drug susceptibility testing (DST) and enrolled at the DR-TB treatment center of Hunan Chest Hospital from 2013 to 2018. Controls were all Drug Susceptible TB (DS-TB) patients confirmed by DST and enrolled at the same hospital during the same period. A multivariable logistic regression model was fitted to identify factors significantly associated with DR-TB.

**Results:**

A total of 17,808 patients (15,534 DS-TB controls and 2274 DR-TB cases) were included in the study, with a mean age of 42.5 years (standard deviation (SD) ± 17.5 years) for cases and 46.1 years (SD ± 19.1 years) for controls. Age 15-64 years (Adjusted odds ratio (AOR = 1.5, 95% CI; 1.4, 1.8)), ethnic minorities (AOR = 1.5; 95% CI; 1.4, 1.8), and a history of previous TB treatment (AOR) = 1.84; 95% CI: 1.57, 2.15) was significantly associated with DR-TB. Being resident in a province outside Hunan was also a significant risk factor (AOR = 1.67; 1.27, 2.21) for DR-TB.

**Conclusion and recommendations:**

To prevent the occurrence of DR-TB in Hunan Province, interventions should be targeted at high-risk demographic groups such as ethnic minorities, individuals of productive age, and residents living outside the province. Interventions must also be targeted to previously treated cases, suggesting the appropriateness of diagnosis, treatment, and follow-up. Understanding the risk factors at the province level helps design strategies for controlling DR-TB due to variations by socioeconomic differences, quality of health care, and healthcare access.

## Introduction

Drug-resistant tuberculosis (DR-TB) is defined as tuberculosis (TB) that is resistant to at least one first-line anti-TB drug (Isoniazid, Rifampicin, Ethambutol, Pyrazinamide, and Streptomycin or any second-line drugs or second-line drugs (including fluoroquinolones and aminoglycosides) or both [1]. DR-TB can occur due to genetic mutations or drug-resistant strains’ transmission [2].

The global burden of DR-TB has increased in the last two decades, with nearly half a million people developing DR-TB every year [1]. It becomes a major challenge to achieving the global End-TB Strategy targets [3]; according to the 2022 WHO global TB report, there were an estimated 450,000 DR-TB cases in 2021 [4]. Nearly half of DR-TB cases were reported in India (26%), the Russian Federation (8.5%), and Pakistan (7.9%) [4].

The Western Pacific region has the third highest burden of TB among WHO global regions (accounting for 18% of cases), next to South-East Asia (43%) and Africa (25%) regions. About 86-90% of the global DR-TB burden is from 30 high-DR-TB burden countries [4], and China is among the 30 high-DR-TB burden countries globally. A study in Huna province among pulmonary TB cases showed that 40.9% of patients were resistant to at least one of the first-line anti-TB drugs [5].

Clinical factors such as a previous history of TB treatment and comorbidities, including Diabetics Mellitus, Human Immunodeficiency Virus infection, and Chronic Obstructive Pulmonary Disease, were identified as risk factors of DR-TB [6]. A systematic review showed that factors such as baseline smear positivity, interruption of treatment, non-adherence, having an adverse drug reaction, being aged > 40 years, being unemployed, and lack of health insurance were significantly associated with the occurrence of DR-TB [7]. Moreover, a contact history with a known TB patient and a previous history of TB treatment failure was associated with a higher risk of developing DR-TB [8]. However, evidence on risk factors of DR-TB in high burden settings such as Hunan province is limited.

Despite significant investments in TB control and treatment strategies in China, TB and DR-TB remain major public health threats in Hunan Province with an increasing disease burden in recent years. Identifying risk factors of DR-TB may aid in designing effective control strategies in the province and other endemic areas. Therefore, this study aims to identify clinical and demographic risk factors associated with DR-TB in Hunan Province.

## Methods

### Study area

This study was conducted in Hunan province, located in South-Central China. Hunan is China’s 7th most populous province with more than 66 million people in 2020 [9]. Hunan Chest Hospital is the largest chest hospital in Hunan Province, located in Changsha City, the province’s capital. It has more than 600 beds and provides services for diagnosing and treating TB and DR-TB. The hospital’s DR-TB treatment center was established in 2011.

### Study design and data sources

A case-control study was conducted to identify the risk factors of DR-TB. All patients diagnosed with DS-TB and DR-TB (Pulmonary vs extrapulmonary, childhood vs adult, bacteriologically confirmed vs clinically diagnosed, and new vs previously treated) in Hunan Chest Hospital from 2013 to 2018 were included in the study. Cases were all DR-TB patients who were confirmed by culture and Drug Susceptibility Testing (DST), enrolled at the DR-TB treatment center of Hunan Chest Hospital, from 2013 to 2018. Controls were all DS-TB patients who were confirmed by DST and enrolled at the same hospital during the same period. Data were obtained from an internet-based TB management information system TB control institute of Hunan province, DR-TB medical records, and DST registration books. Information obtained from the different data sources is linked using patients’ identification. DR-TB is among the notifiable diseases in China, routinely collected by health professionals and notified to the Chinese Center for Disease Control and Prevention (CDC). All DST- and DR-TB patients registered from 2013 to 2018 were included in the analysis. However, patients with unknown drug susceptibility (DST-TB vs DR-TB) status were excluded from the study.

### DS-TB and DR- TB diagnosis

Hunan’s TB institutions follow WHO recommendations for TB diagnosis; using sputum smear microscopy, clinical assessment based on symptoms, sputum culture, and molecular detection. There are 131 counties in Hunan province and only 32 counties can provide comprehensive diagnostic services including culture. In Hunan Chest Hospital, drug susceptibility testing (DST) is mainly carried out in the diagnosis of DR-TB. As a result, sputum specimens from all culture-positive TB patients from throughout the province are referred to the Hunan Chest Hospital for DST. In the hospital, phenotypic DST based on solid and liquid culture techniques, and molecular methods using line probe assays as well as Xpert® MTB/RIF are performed. At the Hunan Chest Hospital, DST is performed for rifampicin, isoniazid, ethambutol, streptomycin, kanamycin, and ofloxacin. Solid and liquid culture are used for the follow-up of patient’s progress and treatment outcomes.

### Variables

The dependent variable of the study was DR-TB status (i.e., DR-TB as cases and DS-TB as controls). DR-TB was defined as resistance to at least one first-line or secondline TB drugs. The independent variables were demographic factors such as age, sex, occupational status, residence type, ethnicity, year of enrolment, and institution type where the diagnosis was made as well as clinical factors such as history of TB treatment and registration type.

### Data analysis

A bivariable logistic regression model was fitted and variables with a *P*-value < 0.2 were selected for inclusion in a multivariable model, to identify factors significantly associated with DR-TB. Adjusted odds ratios (AOR) with 95% confidence intervals (CI) were calculated to quantify the association between the dependent and independent variables and a *p*-value of < 0.05 was used to declare statistical significance. The data were analysed using Stata version 17 software.

## Results

### Sociodemographic characteristics

**Fig. 1:**
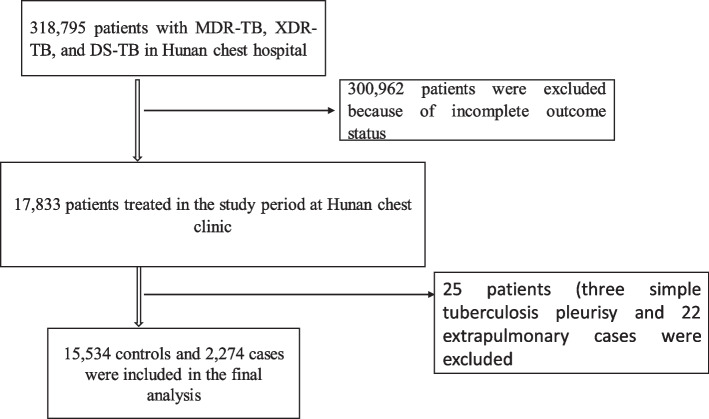
Flowchart of patient record selection process

In Hunan Chest Hospital a total of 318,795 patients were registered for Tuberculosis (TB) between 2013 and 2018. Of these, 300,962 patients were excluded from the analysis due to their unknown outcome status. Moreover, an additional 25 participants (three simple tuberculosis pleurisy and 22 extrapulmonary cases) were excluded. Finally, a total of 17,808 (15,534 DS-TB controls and 2274 DR-TB cases) were included in the final analysis. The remaining (Fig. 1). The mean age of respondents was 42.5 with a standard deviation (SD) of 17.5 for cases and 46.1 years with an SD of 19.1 years for controls. Slightly lower than a quarter of cases (23.9%) and nearly a quarter of controls (26.0%) were female. Around one-fifth (*n* = 448, 19.7%) of cases and controls (*n* = 2868, 18.5%) were under the age of 25 years. Most cases (*n* = 2130, 93.7%) and controls (*n* = 14,868, 95.7%) were Han by ethnicity. The majority, 77.1% (*n* = 1753) of cases and 77.7% (*n* = 12,066) of controls were farmers by occupation. Most participants, including 2092 (92.0%) cases and 14,523 (93.5%) controls, were from Hunan province. The majority, 1813 (79.9%) cases, and 12,379 (80.6%) controls were diagnosed by the Centre for Disease Control. A previous history of TB treatment was reported in 614 (27.0%) cases and 2084 (13.4%) controls (Table 1).

**Table 1:** Socio-demographic and clinical characteristics of study participants in Hunan Province, China, 2013 to 2018

Variables	Total	DR-TB	DS-TB
	N (%)	N (%)	N (%)
**Sex**			
Female	4579 (25.7)	543 (23.9)	4036 (26.0)
Male	13,229 (74.3)	1731 (76.1)	11,498 (74.0)
**Age (years)**			
<=24	3316 (18.6)	448 (19.7)	2868 (18.5)
25-44	4260 (23.9)	703 (30.9)	3557 (22.9)
45-64	7242 (40.7)	888 (39.1)	6354 (40.9)
> = 65	2990 (16.8)	235 (10.3)	2755 (17.7)
**Year**			
2013	703 (3.9)	96 (4.2)	607 (3.9)
2014	991 (5.6)	131 (5.8)	860 (5.5)
2015	2431 (13.7)	298 (13.1)	2133 (13.7)
2016	2712 (15.2)	514 (22.6)	2198 (14.1%)
2017	3320 (18.6)	477 (21.0)	2843 (18.3)
2018	7651 (43.0)	758 (33.3)	6893 (44.5)
**Ethnicity**			
Han majority	16,998 (95.5)	2130 (93.7)	14,868 (95.7)
Other minorities^a^	810 (4.5)	144 (6.3)	666 (4.3)
**Occupation**			
Children and students	647 (3.6)	74 (3.3)	573 (3.7)
Government employees	379 (2.2)	49 (2.2)	330 (2.1)
Farmers and migrants	13,819 (77.6)	1753 (77.1)	12,066 (77.7)
Housekeepers, housework, and unemployed	1252 (7.0)	195 (8.5)	1057 (6.8)
Private employee	341 (1.9)	32 (1.4)	309 (2.0)
Retired	892 (5.0)	104 (4.6)	788 (5.1)
Others^b^	478 (2.7)	67 (2.9)	411 (2.6)
**Type of resistant**			
Primary resistant	15,110 (84.8)	1660 (73)	13,450 (86.6)
Secondary resistant	2698 (15.2)	614 (27)	2084 (13.4)
**Residency status**			
Foreign nationality	584 (3.3)	62 (2.7)	522 (3.4)
Hunan Province	16,615 (93.3)	2092 (92.0)	14,523 (93.5)
Another province	609 (3.4)	120 (5.3)	489 (3.1)
**Detainees**			
No	17,754 (99.7)	2265 (99.6)	15,489 (99.7)
Yes	54 (0.3)	9 (0.4)	45 (0.3)
**Current Diagnosis institution type**			
CDC	14,192 (80.5)	1813 (79.9)	12,379 (80.6)
General Hospital	2904 (16.3)	425 (18.7)	2479 (16.1)
Others^c^	524 (4.0)	30 (1.4)	494 (3.3)
**Patient source**			
Close contact and healthy cheek	48 (0.3)	4 (0.2)	44 (0.3)
Recommend consultation due to symptoms	195 (1.1)	27 (1.2)	168 (1.1)
Referral	6138 (34.5)	733 (32.2)	5405 (34.8)
Seeking consultation due to symptoms	5213 (29.3)	657 (28.9)	4556 (29.3)
Tracing	6178 (34.7)	837 (36.8)	5341 (34.4)
Others	36 (0.2)	16 (0.7)	20 (0.1)
**Type of TB**			
Smear negative	2069 (11.6)	305 (13.4)	1764 (11.4)
Smear positive.	14,702 (82.6)	1870 (82.2)	12,832 (82.6)
Unknown/not recorded	1037 (5.8)	103 (4.4)	959 (6.0)
**Treatment category**			
Initial treatment	15,110 (84.8)	1660 (73)	13,450 (86.6)
Re-treatment	2698 (15.2)	614 (27)	2084 (13.4)
**Total**	17,808 (100)	2274 (12.8)	15,534 (87.2)

**Government employees:** teachers, health care workers, civil servants

**Farmers and migrants**: farmers, workers, migrants, and herdsmen

**Private employees**: catering and food industry workers, commercial service workers, fishermen, sailors, and long-distance drivers

***CDC*** Centre for Disease Control

**Other minorities** ^a^ Dong, Miao, Tujia, Yao, Bai, Buyi, Dai, Gelao, Hani, Hui, Jingpo, Kazakh, Kirgiz, Korean, Lahu, Li, Lisu, Manchu, Mongolian, Salar, She, Tibetan, Tu, Uighur, Wa, Yi, Zhuang

**Other** ^**b**^ Child-care worker and babysitter, and unknown occupation

**Other** ^**c**^ specialist hospital, TB dispensary, township hospital

**NB Percentages are rounded to one-digit decimal place**

### Risk factors for DR-TB in Hunan, China

In the bivariable analysis, age, gender, residence, occupation, ethnicity, and treatment category were selected using a *p*-value threshold of < 0.2. Current residency status, age, ethnicity, and treatment category were statistically significant in the multivariable analysis at a p-value of < 0.05. Occupation was removed in the multivariable analysis due to multicollinearity. The odds of DR-TB among productive age groups (i.e., 15-64 years) were nearly 50% higher than under 15 and elderly (> = 65) populations (AOR = 1.5; 95% CI: 1.4, 1.8). Other ethnic minorities had nearly 50% higher odds of developing DR-TB than Han majorities (AOR = 1.5; 95% CI: 1.2, 1.8). The odds of DR-TB among residents of neighboring provinces were significantly higher compared with those of Hunan residents (AOR = 1. 7; 95% CI: 1.4, 2. 1). Similarly, the odds of DR-TB in previously treated cases were nearly two times higher than in the new treatment category (AOR = 2.3; 95% CI: 2.1, 2.6). The Hosmer and Leme show goodness of fit test showed the model was adequately fitted (*P*-value = 0.12) (Table 2).

**Table 2:** Factors associated with DR-TB in Hunan, China

Variables	Outcome		COR with 95% CI	AOR with 95% CI
	Cases	Control		
**Age (in years)**				
< 15 and ≥ 65	408	3903	1	1
15-64	1866	11,631	1.5 (1.4, 1.7)	1.5 (1.4, 1.8)
**Gender**				
Female	543	4036	1	1
Male	1731	11,498	1.2 (1.0, 1.2)	1.1 (0.9, 1.2)
**Ethnicity**				
Han majority	2130	14,868	1	1
Other minorities^a^	144	666	1.5 (1.3, 1.8)	1.5 (1.2, 1.8)
**Residency status**				
Hunan province	2092	14,523	1	
Foreign nationality	62	522	0.8 (0.6, 1.1)	0.8 (0.6, 1.1)
Another province	120	489	1.7 (1. 4, 2.1)	1.7 (1.4, 2.1)
**Treatment history**				
New Patients	1660	13,450	1	1
Previously treated patients	614	2084	2.4 (2.1, 2.6)	2.3 (2.1, 2.6)
**Occupation**				
Farmers and migrants	1753	12,066	1	1
Others^b^	521	3468	1.0 (0.9-1.1)	1.2 (1.1-1.3)
**Year at enrolment**				
2013-2015	525	3600	1	NA
2016-2018	1749	11,934	1.0 (0.9, 1.1)	

**Other minorities** ^**a**^ Dong, Miao, Tujia, Yao, Bai, Buyi, 3 = Dai, Gelao, Hani, Hui, Jingpo, Kazakh, Kirgiz, Korean, Lahu, Li, Lisu, Manchu, Mongolian, Salar, She, Tibetan, Tu, Uighur, Wa, Yi, Zhua

**Other Occupation** ^b^ **:** Children, students, government employees (teachers, health care workers, cadre/civil servants), housekeepers, housework, unemployed, private employees (catering and food industry, waiting for people in a public place, commercial service, fisherman, /boat people, seaman, and long-distance driver), and retired

**Farmers and migrants**: farmers, workers, migrants, and herdsmen

***NA*** Not eligible for the multivariable analysis

## Discussion

This case-control study identified risk factors of DR-TB in Hunan province. Clinical factors such as a previous history of TB treatment were identified as a significant risk factor for DR-TB (relative to DS-TB). Moreover, demographic factors such as ethnic minorities, productive age group, and residency status outside Hunan province were identified as risk factors for DR-TB.

The finding of the previous history of TB treatment to be associated with a higher risk of DR-TB is consistent with previous studies conducted in China [10, 11], India [12], a multicenter study from eight countries [13], Pakistan [14, 15], Brazil [16, 17], Northwest Ethiopia [18–20], United Kingdom [10], Saudi Arabia [21] and South Africa [22]. The current finding is also in agreement with a systematic review and meta-analysis conducted in Europe [23], and a global systematic review and meta-analysis [24].

Ethnic minorities were at a higher odd of developing DR-TB compared with the Han majority. The finding agrees with studies conducted to assess health inequality in Europe and the United States, which showed that ethnic minorities had poorer health outcomes than ethnic majorities [25–27]. Possible reasons for this discrepancy may include poor treatment adherence to TB treatment and significant ethnic health inequalities arising from the interplay between ethnicity, spatial disparities, and socioeconomic status among different ethnic groups [26].

Productive age groups (i.e.,15-64 years) were at a higher risk of experiencing DR-TB. This finding is in line with a multicenter study conducted in South Korea [28], Brazil [17], Bangladesh [29], China [30], and United States [30]. The second possible justification could be productive population group was at a higher risk of poor treatment adherence or interruption of TB treatment due to work or travel [31].

In this study, residency outside of Hunan province was positively associated with DR-TB risk. This finding is in line with a study conducted in Hong Kong [31]. The possible reason could be residents from other provinces are classified as internal migrants according to the hukou system, a household registration system in China that permits a permanent resident for a single address issued by the government of China to each family member given at birth or by employment in the formal sectors [32]. The hukou system allows each family member to have essential access to public services, education, health services, social benefits, and job recruitment by the governmental or private sectors [33].

However, inconsistent evidence has been found concerning residency status, with some previous studies showing that residents from permanent residents were at a higher risk of developing DR-TB compared with residents from another province [34]. Similarly, a study conducted in Shanghai, China among migrants and permanent residents revealed that the transmission rate of DR-TB was higher among permanent residents than temporary migrants and residents from the local province [35].

The difference would be the higher population density in Shanghai (21,700/km^2^) [36] compared with the population density in Hunan province (326.27/km^2^) [37]. Moreover, the high cost of rent in Shanghai results in living in a condensed environment that further increases the risk of developing primary resistance [36]. Furthermore, most migrants were from rural areas, which are characterized by less cost of life and less condensed areas that will decrease the risk of acquiring drug-resistant tuberculosis.

While this study identified individual-level risk factors associated with DR-TB in Hunan province using a large sample size, it has several limitations. First, as the study was conducted using secondary data, possible important determinants such as behavioral factors (e.g., cigarette smoking and alcohol drinking) and clinical factors including adherence, baseline smear status, and time of culture conversion were not assessed. Moreover, due to the nature of the study design (i.e., hospital-based case-control study) selection bias might have been introduced. For instance, the study lacks generalizability to the general population as cases and controls were drawn purposively from the hospital. Of 318,795 TB patients, only 17,831 had DST result and were included in the study. As a result, the finding lacks external validity and will not be generalizable to TB patients in Hunan Province. The study may also be liable to recall bias and some misclassifications in the exposure and outcome status. The study also lacks drug-resistant profile of patients due to the secondary nature of the data.

The clinical burden of DR-TB is increasing over time worldwide and most countries have not achieved the END-TB WHO targets. Even though some studies on determinants of DR-TB have been conducted, the evidence on risk factors remains limited, particularly in Hunan province and other parts of China. The current study provides some clues as to the most important risk factors for DR-TB in Hunan province. The risk factors we identified are potential targets for intervention – for example, healthcare providers could focus more on patients having a previous history of TB treatment.

## Conclusion

To prevent the occurrence of DR-TB in Hunan Province, interventions should be targeted at high-risk demographic groups such as individuals in productive age, ethnic minorities, and residents living outside the province. Moreover, interventions must also be targeted to previously treated cases, suggesting the appropriateness of diagnosis, treatment, follow-up, and monitoring. Understanding risk factors of DR-TB in Hunan Province, China is important in designing strategies for controlling DR-TB due to variations of health care access, socio-economic variations, and quality health care. Strengthening the existing surveillance system and improving health care access are required for proper diagnosis and treatment of DR-TB.

## Data Availability

Data will be available upon request from the corresponding author.

## Abbreviation

AOR: Adjusted Odds Ratio
CDC: Centre for Disease Control
CI: Confidence Interval
DR-TB: Drug-resistant Tuberculosis
DST: Drug Susceptibility Testing
DS-TB: Drug Susceptibility Tuberculosis
INH: Isoniazid
R: Rifampicin
SD: Standard Deviation
TB: Tuberculosis
US: United States
WHO: World Health Organization

## References

[1] World Health Organization (2021). Global tuberculosis report.

[2] David HL (1970). Probability distribution of drug-resistant mutants in unselected populations of mycobacterium tuberculosis. Appl Microbiol.

[3] WHO (2008). Global Tuberculosis Control SURVEILLANCE, PLANNING, FINANCING.

[4] World Health Organization (2022). Global tuberculosis report.

[5] Zhao L-l, Chen Y, Chen Z-n, Liu H-c, Hu P-l, Sun Q (2014). Prevalence and molecular characteristics of drug-resistant mycobacterium tuberculosis in Hunan, China. Antimicrob Agents Chemother.

[6] Rumende CM (2018). Risk factors for multidrug-resistant tuberculosis. Acta Med Indones.

[7] Pradipta IS, Forsman LD, Bruchfeld J, Hak E, Alffenaar JW, Baya B (2018). Risk factors of multidrug-resistant tuberculosis: A global systematic review and meta-analysis Clinical risk factors associated with multidrug-resistant tuberculosis (MDR-TB) in Mali. J Inf Secur.

[8] Baya B, Achenbach CJ, Kone B, Toloba Y, Dabitao DK, Diarra B (2019). Clinical risk factors associated with multidrug-resistant tuberculosis (MDR-TB) in Mali. Int J Infect Dis.

[9] https://en.wikipedia.org/wiki/List_of_Chinese_administrative_divisions_by_population: .

[10] Mohd Shariff N, Shah SA, Kamaludin F (2016). Previous treatment, sputum-smear nonconversion, and suburban living: the risk factors of multidrug-resistant tuberculosis among Malaysians. Int J Mycobacteriol.

[11] Zhang C, Wang Y, Shi G, Han W, Zhao H, Zhang H (2016). Determinants of multidrug-resistant tuberculosis in Henan province in China: a case control study. BMC Public Health.

[12] Ladha N, Bhardwaj P, Chauhan NK, Naveen KHS, Nag VL, Giribabu D. Determinants, risk factors and spatial analysis of multi-drug resistant pulmonary tuberculosis in Jodhpur, India. Monaldi Arch Chest Dis. 2022;92(4) 10.4081/monaldi.2022.2026 35044136

[13] Dalton T, Cegielski P, Akksilp S, Asencios L, Campos Caoili J, Cho SN (2012). Prevalence of and risk factors for resistance to second-line drugs in people with multidrug-resistant tuberculosis in eight countries: a prospective cohort study. Lancet.

[14] Ahmad AM, Akhtar S, Hasan R, Khan JA, Hussain SF, Rizvi N (2012). Risk factors for multidrug-resistant tuberculosis in urban Pakistan: a multicenter case-control study. Int J Mycobacteriol.

[15] Ullah I, Javaid A, Tahir Z, Ullah O, Shah AA, Hasan F (2016). Pattern of drug resistance and risk factors associated with development of drug resistant mycobacterium tuberculosis in Pakistan. PLoS One.

[16] Migliori GB, Tiberi S, Zumla A, Petersen E, Chakaya JM, Wejse C (2020). MDR/XDR-TB management of patients and contacts: Challenges facing the new decade. The 2020 Clinical update by the global tuberculosis network. Int J Infect Dis.

[17] Jacobs MG, Pelissari DM, Pinto VL (2018). Factors associated with the drug-resistant tuberculosis incidence rate in Brazil. Int J Tuberc Lung Dis.

[18] Alene KA, Viney K, McBryde ES, Gray DJ, Melku M, Clements ACA (2019). Risk factors for multidrug-resistant tuberculosis in Northwest Ethiopia: a case-control study. Transbound Emerg Dis.

[19] Eshetie S, Gizachew M, Dagnew M, Kumera G, Woldie H, Ambaw F (2017). Multidrug resistant tuberculosis in Ethiopian settings and its association with previous history of anti-tuberculosis treatment: a systematic review and meta-analysis. BMC Infect Dis.

[20] Badgeba A, Shimbre MS, Gebremichael MA, Bogale B, Berhanu M, Abdulkadir H (2022). Determinants of multidrug-resistant mycobacterium tuberculosis infection: a multicenter study from southern Ethiopia. Infect Drug Resist.

[21] Alrajhi AA, Abdulwahab S, Almodovar E, Al-Abdely HM (2002). Risk factors for drug-resistant mycobacterium tuberculosis in Saudi Arabia. Saudi Med J.

[22] Andrews JR, Shah NS, Weissman D, Moll AP, Friedland G, Gandhi NR (2010). Predictors of multidrug- and extensively drug-resistant tuberculosis in a high HIV prevalence community. PLoS One.

[23] Faustini A, Hall AJ, Perucci CA (2006). Risk factors for multidrug resistant tuberculosis in Europe: a systematic review. Thorax..

[24] Pradipta IS, Forsman LD, Bruchfeld J, Hak E, Alffenaar JW (2018). Risk factors of multidrug-resistant tuberculosis: a global systematic review and meta-analysis. J Inf Secur.

[25] Bleich SN, Jarlenski MP, Bell CN, LaVeist TA (2012). Health inequalities: trends, progress, and policy. Annu Rev Public Health.

[26] Blom N, Huijts T, Kraaykamp G (2016). Ethnic health inequalities in Europe. The moderating and amplifying role of healthcare system characteristics. Soc Sci Med.

[27] LaVeist TA, Lebrun LA (2010). Cross-country comparisons of racial/ethnic inequalities in health. J Epidemiol Community Health.

[28] Lee EG, Min J, Kang JY, Kim SK, Kim JW, Kim YH (2020). Age-stratified anti-tuberculosis drug resistance profiles in South Korea: a multicenter retrospective study. BMC Infect Dis.

[29] Rifat M, Milton AH, Hall J, Oldmeadow C, Islam MA, Husain A (2014). Development of multidrug resistant tuberculosis in Bangladesh: a case-control study on risk factors. PLoS One.

[30] Zhao Y, Xu S, Wang L, Chin DP, Wang S, Jiang G (2012). National survey of drug-resistant tuberculosis in China. N Engl J Med.

[31] Law W, Yew W, Chiu Leung C, Kam K, Tam C, Chan C (2008). Risk factors for multidrug-resistant tuberculosis in Hong Kong. The international journal of tuberculosis and lung disease.

[32] Song Q, Smith JP (2019). Hukou system, mechanisms, and health stratification across the life course in rural and urban China. Health Place.

[33] Kuang L, Liu L (2012). Discrimination against rural-to-urban migrants: the role of the hukou system in China. PLoS One.

[34] Lecai J, Mijiti P, Chuangyue H, Mingzhen L, Qian G, Weiguo T (2021). Predictors and trends of MDR/RR-TB in Shenzhen China: a retrospective 2012–2020 period analysis. Infection and Drug Resistance.

[35] Ge E, Li D, Luo M, Tsui K, Waye M, Shen X (2018). Transmission of multidrug-resistant tuberculosis in Shanghai: roles of residential status. The International Journal of Tuberculosis and Lung Disease.

[36] Lai P-C, Low CT, Tse WS, Tsui CK, Lee H, Hui PK (2013). Risk of tuberculosis in high-rise and high density dwellings: an exploratory spatial analysis. Environ Pollut.

[37] Hunan population density: https://en.wikipedia.org/wiki/Hunan:.

